# Genetic Variation of the Host Plant Species Matters for Interactions with Above- and Belowground Herbivores

**DOI:** 10.3390/insects5030651

**Published:** 2014-08-29

**Authors:** Dinesh Kafle, Andrea Krähmer, Annette Naumann, Susanne Wurst

**Affiliations:** 1 Collaborative Research Center (CRC) 973, Institute of Biology, Functional Biodiversity, Freie Universität Berlin, Königin-Luise-Str. 1-3, Berlin 14195, Germany; E-Mail: s.wurst@fu-berlin.de; 2 Julius Kühn-Institut, Federal Research Centre for Cultivated Plants, Institute for Ecological Chemistry, Plant Analysis and Stored Product Protection, Königin-Luise-Str. 19, Berlin 14195, Germany; E-Mails: andrea.kraehmer@jki.bund.de (A.K.); anauman1@gwdg.de (A.N.)

**Keywords:** above-belowground herbivores, genotype, C/N ratio, PI, FT-IR spectroscopy

## Abstract

Plants are challenged by both above- and belowground herbivores which may indirectly interact with each other via herbivore-induced changes in plant traits; however, little is known about how genetic variation of the host plant shapes such interactions. We used two genotypes (M4 and E9) of *Solanum dulcamara* (Solanaceae) with or without previous experience of aboveground herbivory by *Spodoptera exigua* (Noctuidae) to quantify its effects on subsequent root herbivory by *Agriotes* spp. (Elateridae). In the genotype M4, due to the aboveground herbivory, shoot and root biomass was significantly decreased, roots had a lower C/N ratio and contained significantly higher levels of proteins, while the genotype E9 was not affected. However, aboveground herbivory had no effects on weight gain or mortality of the belowground herbivores. Root herbivory by *Agriotes* increased the nitrogen concentration in the roots of M4 plants leading to a higher weight gain of conspecific larvae. Also, in feeding bioassays, *Agriotes* larvae tended to prefer roots of M4 over E9, irrespective of the aboveground herbivore treatment. Fourier-Transform Infrared Spectroscopy (FT-IR) documented differences in metabolic profiles of the two plant genotypes and of the roots of M4 plants after aboveground herbivory. Together, these results demonstrate that previous aboveground herbivory can have genotype-specific effects on quantitative and qualitative root traits. This may have consequences for belowground interactions, although generalist root herbivores might not be affected when the root biomass offered is still sufficient for growth and survival.

## 1. Introduction

Both above- and below-ground plant parts are attacked by a wide array of aboveground (AG) and belowground (BG) herbivores. Along with this direct interaction with the plant, these spatially and in some cases temporally separated herbivores interact with each other indirectly via their shared host plant [1,2]. The interactions are mediated by changes in plant traits such as quantitative and qualitative changes in plant primary and secondary metabolites [3,4,5] and plant growth. Such interactions might be beneficial, detrimental or neutral to each other, largely depending upon the defense strategy of the plant species upon herbivory, the feeding guild, the modes of feeding, the sequence of arrival of the herbivores, and abiotic factors [5,6,7,8,9,10]. In all previous studies on the indirect interaction between AG and BG herbivores, both types of herbivores were added to the plant system either simultaneously or sequentially, and interacted with the plant at least partly at the same time. However, past interactions with AG or BG herbivores may change plant traits with subsequent effects on future plant–herbivore interactions. Thus, in our experiment, we used a novel approach and introduced the BG herbivores to the root system after removal of AG herbivores from the plants to test for history effects of AG herbivory mediated by changes in plant traits.

One major plant response upon herbivory is the local or systemic expression of resistance traits such as induction of defensive compounds in order to reduce herbivory and increase plant fitness [11,12]. These changes may also affect the interactions between herbivores feeding on spatially separated plant parts of the same plant [5,13]. In an experiment with cotton plant, it was found that BG herbivores (wireworms: *Agriotes lineatus* larvae) decreased the performance of AG herbivores (Beet armyworm: *Spodoptera exigua*) by increasing the foliar terpenoid levels, while no effects were found on wireworm performance due to AG herbivores [14]. In another experiment, Kaplan *et al.* [4] found that AG herbivores (*Manduca sexta*) increased the performance of root parasitic nematodes in tobacco (*Nicotiana tabacum)* by relocating photoassimilates from shoot to the root in response to feeding damage, while nematode enhanced the performance of AG herbivores by interfering with the biosynthesis of the alkaloid nicotine in the root, which is transported to the leaves upon herbivory. 

As an alternative to induction of costly resistance traits, plants also employ tolerance responses to deal with herbivory stress [15]. Such responses include increased photosynthetic and growth rates, increased tillering, and reallocation of resources [16]. Both resistance and tolerance responses may co-occur in the same plant species [17]. Plants relocate their valuable resources to other compartments and tissues which are not under the threat of herbivory. This way, they can protect valuable nutrients and use them for later growth and development, while the low nutritional status of the feeding site leads to poor performance of the herbivores [4,18]. Such “herbivory-induced resource sequestration” [19] may also alter the plant mediated interaction between AG and BG herbivores. For example, Johnson *et al.* [20] used barley plants to analyze the plant nutrients-mediated interaction between AG and BG herbivores, and found that root-feeding wireworms promoted AG aphid numbers by increasing essential amino acids in the leaves, while aphids enhanced wireworm growth by increasing the concentration of root minerals such as nitrogen (N), sulphur (S), calcium (Ca), phosphorus (P) and potassium (K).

Most of the previous studies on indirect interactions among herbivores that share a common host plant focused on AG herbivores and AG plant parts (e.g., [21]). The other half of the plant system, *i.e.*, the root, has received far less consideration in ecological research, probably because of the difficulties associated with the observation and analysis of BG interactions. However, the root is also an integral part of the plant system which serves several vital functions such as providing water and nutrients to the shoots. Apart from these basic functions, roots play a pivotal role in plant defense and growing evidence suggests that induction of plant defense in spatially separated AG and BG plant parts occurs [5,13,14,17,22]. In the majority of plant species which have been studied for defense compounds, the root tissue contains similar defensive compounds as present in the aboveground parts and may thereby be able to defend itself against root herbivores [23]. However, the root may not be as efficient as leaves in the defense response against herbivores because the perception of herbivory and defense signaling is different in the root system [24]. In some plant species such as tobacco, plant defense compounds such as alkaloids are produced in the root and then transported to the shoot upon herbivory [25]. Roots might be a strategically safe site for the biosynthesis and storage of defense compounds because they are surrounded by soil and out of reach of AG herbivores [4]. As a tolerance response to AG herbivory, the root also provides a temporary storage site for primary plant metabolites [18]. Optimal defense theory (ODT) predicts that the plant customizes its defense to the plant parts of high risk and greatest value but it ignores the possibility of such a fine-tuned defense strategy of the plant to protect the root tissue because it is considered less valuable to the plant and less vulnerable to herbivores than the shoot [17]. However, as already mentioned, the importance of the root in tolerance and defense response has already been documented, though there are fewer studies. 

Genetic variation of the host plant species enables plants to cope with a multitude of herbivores with their varying resistant traits, and these genetic variations also shape the herbivore community [26,27]. Genetic variation of the plant species may also alter relative competitive strengths among herbivore species thereby promoting their co-existence on individual plants [28]. Some studies have examined whether genetic variation of the plant populations also adds specificity in plant–insect interactions, but such studies mainly focused on AG herbivores only [29,30,31]. So far, only few studies have reported effects of genetic variation in plants on herbivore-induced changes in secondary compounds having significant impacts on interactions between AG and BG herbivores [32,33].

To investigate the impact of previous feeding by AG herbivores on plant and BG herbivore performance, and to see if the genetic variation of a plant species mediates the interactions, we carried out a greenhouse experiment using bittersweet nightshade *Solanum dulcamara* L. (Solanaceae) as a model plant. *S. dulcamara* is a common perennial vine plant species of Eurasian region which grows in a wide range of habitats from swamp to dry sand dunes [34]. Induction of defense compounds like polyphenol oxidase, peroxidases, and protease inhibitor activity upon leaf herbivory has been shown in this plant [35,36]; thus it is considered as a potential new model plant for ecological research. We used two genotypes (M4 and E9) of *S. dulcamara* plants to analyze the effect of previous shoot herbivory by *S. exigua* larvae on the root herbivore *Agriotes* spp. via changes in plant traits. These two genotypes belong to two different habitats; M4 was collected from a habitat surrounded by agricultural fields, while E9 was collected from a natural forest. Both habitats are swampy areas surrounding lakes. The herbivore abundance and identity can be assumed to be different in these two geographical locations, therefore we also expected differential responses of the genotypes to herbivory. We introduced the AG and BG herbivores sequentially with seven days of lag phase in-between; *i.e.*, the BG herbivores were added seven days after the removal of the AG herbivores. Then, we measured plant responses to ask (1) whether previous AG herbivory affects the induction of resistance and tolerance traits of the plant, (2) whether these changes in the plant traits affect the BG herbivores, and (3) whether these effects vary among plant genotypes. Plant responses were examined on the level of their AG and BG biomasses, the carbon (C) and nitrogen (N) concentration, the total protein content and the protease inhibitor activity (PI). For fast estimation of changes in primary and secondary metabolite profiles, leaf and root material was additionally investigated by Fourier-Transform Infrared Spectroscopy (FT-IR).

## 2. Experimental Section 

### 2.1. Model Plant

*S. dulcamara* plants were obtained from two clonal populations maintained in the greenhouse of Freie Universität Berlin (temperature 20/16 °C and 16/8 hour day/night). The greenhouse populations were derived from cuttings of individual *S. dulcamara* plants from two different locations located near Berlin in June 2011; the genotype M4 from Mehrow (Wendtsee, Germany; 52° 25' 7.15'' N, 13° 46' 27.87'' E) and the genotype E9 from Erkner (Wupatzsee, Germany; 52° 34' 7.07'' N, 13° 38' 3.43'' E). Cuttings with two nodes and a length of about 7–8 cm were obtained from the shoots of the clonal populations and grown in plastic pots (13 cm diameter, 11.3 cm height) containing 850 mL of soil: sand mixture. The soil was taken from the research site of Freie Universität Berlin at Albrecht-Thaer-Weg 6, and mixed with sand in the ratio of 3:1 (soil: sand by volume). The mixture was steamed for 3 hours at 90 °C to exclude root herbivores. The top layer of the soil was covered with sand grit to prevent the growth of green algae and infestation of *fungus gnats* (Sciaridae). Pots were placed on individual plastic plates and randomized weekly to homogenize for variances due to abiotic factors such as light conditions. 

### 2.2. Study Organisms

Generalist root herbivores *Agriotes* spp. (Elateridae) were collected from an abandoned agricultural field in Grossbeeren, Germany, in spring 2013. Due to the destructive nature of species identification, the larvae were not identified to species level, but previous sampling and identification showed that the species *Agriotes obscurus* is predominant in this area [37]. These larvae were kept in petri dishes with wet pieces of paper towel at 4 °C until use. Larvae of the generalist herbivore *Spodoptera exigua* (Noctuidae) were obtained from the laboratory cultures maintained at the facility of Applied Zoology of FU Berlin. They were reared on artificial diet (wheat germ based basic diet and a vitamin mix) in a climate chamber at 24 °C and 70% humidity under 16/8 hour day/night light cycle.

### 2.3. Herbivore Treatment

Five-week-old healthy plants were selected from the genotypes M4 and E9; a total of 52 individuals of each genotype was used. To half of the plants, a second instar *S. exigua* larvae was added in a clip cage and allowed to feed on the eighth leaf (first fully expanded leaf) and on three consecutive leaves for a total of seven days (48 hours on the first three leaves each and 24 hours on the last leaf). The other half of the plants did not receive aboveground herbivory (AGH). After removal of *S. exigua* larvae, the plants were kept without any herbivores for one week before three *Agriotes* larvae were added to the roots of half of the AGH and half of the non-AGH plants for belowground herbivory (BGH). This lag phase was added to see how long the effects of AGH could last in the plant and the duration of lag phase was decided for practical reasons. Average weight of larvae in each individual pot was 75.88 ± 1.14 mg (mean ± SE). In total, there were 13 replicates per treatment (*i.e.*, Control, AGH only, BGH only and AGH+BGH). The plants were treated for one week with the root herbivores and then harvested. Root subsamples were collected, frozen immediately and stored at −80 °C to further analyze protease inhibitors (PIs) and total protein content; the rest of the roots and shoots were dried in an oven at 55 °C for three days before measuring their respective dry biomass. The root herbivores from individual pots were collected and weighed together to analyze the collective weight gain of the larvae in each plant. 

### 2.4. Root Material and Sample Extraction for Total Protein and PI Analysis

The roots of the harvested plants were washed immediately and 175–200 mg of fresh fine root samples were weighed, placed in 1.5 mL Eppendorf tubes, flash frozen in liquid nitrogen and stored at −80 °C. Samples were powdered by grinding them within the Eppendorf tube using FastPrep homogenizer (FastPrep®-24, MP Biomedicals, Santa Ana, CA, USA). To extract the working sample for analysis, 700 µL of extraction buffer (1 L: 0.1 M Tris-Cl, 50 g PVPP, 2 g phenylthiourea, 5 g diethyldithiocarbamate, 18.6 g 0.05 M Na_2_EDTA, pH = 7.6) was added in the Eppendorf tube. Supernatant was collected after centrifuging sample for 20 min at 18,000 g at 4 °C. The supernatant was centrifuged again in the same way to obtain clean supernatant and stored at −20 °C until use. 

### 2.5. Total Protein Content Measurement

Total protein in the root tissue was measured using Bradford protein assay. Bradford staining solution (Roti-Quant, Roth, Germany) was freshly prepared by diluting in the ratio of 1:4 in distilled water. Albumin (Roth, Germany) was dissolved in 0.1 M Tris buffer to a 1 mg/mL stock solution. For references, the albumin stock solution (1 mg albumin [Roth, Karlsruhe, Germany] per mL of 0.1 M Tris buffer) was diluted to concentrations of 0.25, 0.125, 0.0628, 0.033, and 0.017 mg/mL. Samples were defrosted on ice and shortly shaken on the vortex shaker. The samples were diluted 11× using 0.1 M Tris buffer. Then, 10 μL of samples were pipetted into 96 well plates prefilled with 200 μL staining solution. Three technical replicates were run for each sample and each plate contained three Tris buffer blank samples. A micro-Quant plate reader (BioTek Instruments, Inc., Winooski, VT, USA) was used for the photometric measurement and plate reader software KC junior (BioTek Instruments, Inc.) was used for the analysis. According to the reference curve, the total protein content per g FW (Fresh Weight) was calculated. 

### 2.6. Protease Inhibitor Measurement

PI in the root tissue was measured using Serine PI microplate assay after Bode *et al.* [38]. In short, PI activity was measured by mixing 20 µL of reaction buffer (0.1 M TRIS, pH 7.6), 10 µL of 0.25 mg/mL trypsin in 0.1 M TRIS, and 20 µL sample, and incubating at 37.5 °C for 5 min. Then, 20 µL of 3.1 mg/mL N_α_-Benzoyl-DL-arginine β-naphthylamide hydrochloride (BANA) (Sigma-Aldrich, Munich, Germany) in dimethyl sulfoxide (DMSO) (Roth, Karlsruhe, Germany) was added and incubated for another 20 min at 37.5 °C. The reaction was terminated by adding 100 µL of 2% HCl in ethanol and the absorbance at 540 nm was measured as background of each sample. Then, 100 µL of 0.06% *p*-dimethylamino cinnamaldehyde (DACA) (Sigma-Aldrich, Munich, Germany) in ethanol was added and the reaction was allowed to proceed for 30 min at room temperature. As positive control (with trypsin, no sample), soybean trypsin inhibitor standards of six concentrations (0.24, 0.12, 0.06, 0.03, 0.015 and 0.0075 mg/mL in 0.1 M TRIS) were also included on each plate. Finally, the total absorbance was measured at 540 nm. A standard curve derived from soybean trypsin inhibitor standards was used to determine the PI concentration. 

### 2.7. FT-IR Spectroscopy and Sample Preparation

To get a fast overall impression of variations in the metabolic profile due to either the plant genetic background or environmental impacts, Fourier-Transform Infrared (FT-IR) spectroscopy was shown to be a suitable and easily applicable method for non-target analyses [39,40,41,42]. Already on the level of dried and ground leaf material, differences in the concentration of specific compound classes or even individual substances can be observed by characteristic absorption patterns in the spectrum and in combination with chemometric methods like principle component analysis (PCA) or hierarchical cluster analysis [43,44,45]. Hence, leaf and root material of both *Solanum dulcamara* genotypes was analyzed. Plant material was air dried after harvest and ground to fine powder by a ball mill (Mixer Mill MM 400, Retsch GmbH, Haan, Germany). FT-IR spectra were recorded with a Platinum ATR single reflection diamond FT-IR spectrometer (ALPHA Bruker Optics, Ettlingen, Germany) with a spectral resolution of 4 cm^−1^ and 32 scans from 4000–375 cm^−1^ using three subsamples. Mean spectra were created out of the three replicates using the instrument software OPUS 6.5 (Bruker Optics, Ettlingen, Germany).

### 2.8. Chemometrics

For estimating the variability of the samples, principal component analysis (PCA) was performed using the statistic software The Unscrambler^®^ X version 10.0 (CAMO Software, Oslo, Norway).

### 2.9. Carbon and Nitrogen Concentration Measurement

Leaf and root materials were ground in Eppendorf tubes by using a mixer mill (Mixer Mill MM 400, Retsch GmbH, Haan, Germany) and were dried again for at least 24 hours. Leaf and root nitrogen concentration was determined on the dry samples by using an elemental analyser (Euro EA, HEKAtech GmbH, Wegberg, Germany). 

### 2.10. Choice and Non-Choice Feeding Bioassay

To check if the *Agriotes* larvae had a preference for the roots from one treatment over another, choice and non-choice feeding bioassays were carried out. For the non-choice bioassay, 10 ± 1 mg of a dried root sample was taken, rewetted and kept in a petri dish with one *Agriotes* larvae. The *Agriotes* larvae were allowed to feed on the root material for 9 days. Likewise, in the choice bioassay, the larva was provided with 10 ± 1 mg of rewetted root material from each of four treatments from the same genotype in four sides of the petri dish and allowed to feed for 12 days. Both genotypes were tested similarly in parallel, but separately. The bioassay was carried out in a climate chamber (York Deutschland GmbH) at 10 °C and 70% humidity under 16/8 hour day/night light cycle. Then, the total amount of root material eaten and the weight gain of the larvae were measured.

### 2.11. Statistical Analysis

All the statistical analyses were carried out in “R”, version 3.0.0 [46]. One-way and two-way ANOVA were performed to test the significance of the treatments within and between the genotypes. Statistical significance was set at *p* < 0.05. In some analyses, log and square root transformation of raw data were performed in order to meet the assumptions of ANOVA. The data that did not fulfill the assumptions of ANOVA were analyzed with Generalized Linear Models (GLM) assuming gamma distribution of errors. While analyzing the growth of *Agriotes* larvae, all the replicates where the larvae were missing or dead were excluded from the statistical analyses. In the non-choice bioassay, the effects of the treatments on weight gain of *Agriotes* larvae on M4 root were analyzed in generalized least square models (GLS). Non-homogeneity of variances was accounted for by applying the varIdent command [47]. Means and standard errors are reported in the results section.

## 3. Results and Discussion

### 3.1. Results

#### 3.1.1. Herbivore Performance

##### 3.1.1.1. *Spodoptera Exigua*

**Figure 1:**
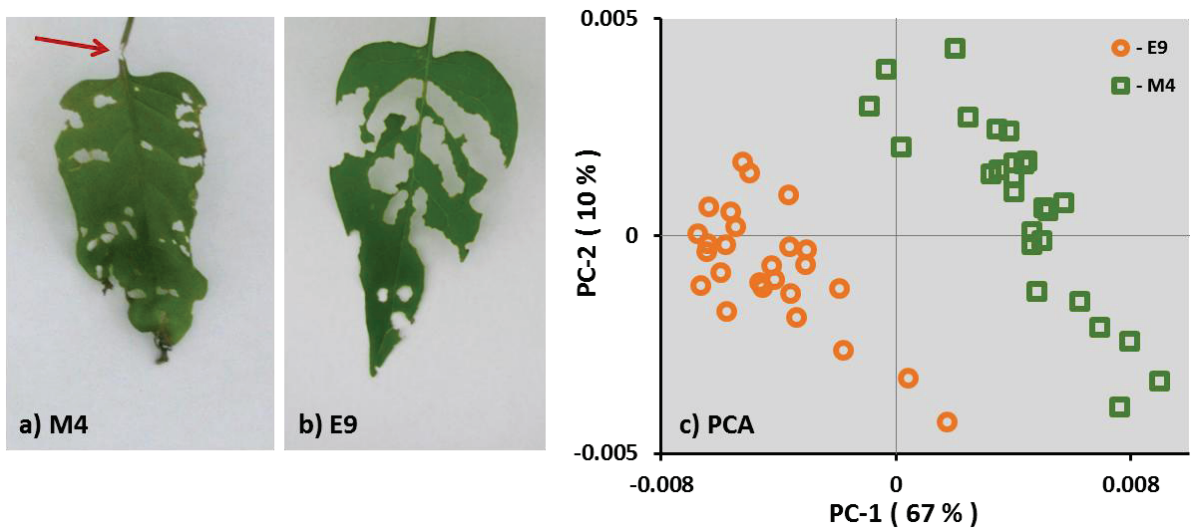
Feeding pattern of *S. exigua* larvae on the leaves of genotype M4 (**a**) and E9 (**b**). The arrow shows a petiole chewed by the larvae in a M4 leaf. Principle component analysis (PCA) of the FT-IR spectra (**c**) of M4 and E9 over the spectral range of 360–375 cm^−1^. Spectra are treated by first derivative and multiple scatter correction.

Feeding behavior of *S. exigua* larvae differed between the examined genotypes. In 65% of the first and second leaves of the genotype M4, it was observed that *S. exigua* larvae chewed the petiole of the leaves thereby inducing the wilting of the leaves (Figure 1a). In contrast, *S. exigua* chewed the petiole of only a few (7%) of the first and second leaves of genotype E9, but fed evenly on the leaf tissue (Figure 1b). There was no mortality of the *S. exigua* larvae, and the weight gain of the larvae was not different on the two genotypes (M4: 156.62 ± 14.99; and E9: 149.33 ± 13.13; mean ± SE).

This different feeding behavior observed on M4 and E9 suggests a different metabolic profile of both genotypes. FT-IR analysis of the dried and ground leaf material supports this hypothesis as shown in the principle component analysis (PCA) in Figure 1c. Samples of both genotypes, M4 and E9 were grouped in individual clusters independently of the previous AG herbivore treatment. This result shows that the two genotypes of *S. dulcamara* differ in metabolic profiles in leaves as well as in roots (see Supplementary Figure S1).

##### 3.1.1.2. *Agriotes* spp. Larvae

There were no significant differences in the weight gain (Table 1) and mortality of the *Agriotes* larvae feeding on the two treatments of both genotypes. In M4 plants, in total four larvae were found dead, one in the BGH and three in AGH+BGH treatment, while only one larva was missing from the AGH+BGH treatment. In E9 plants, only one larva was found dead in the BGH treatment, while two larvae were recovered instead of three in four replicates of the AGH+BGH treatment. 

**Table 1:** Weight gain of *Agriotes* larvae (mean ± SE) in the different treatments.

Genotype	BGH	AGH+BGH
M4	2.00 ± 1.13	0.71 ± 0.93
E9	1.35 ± 1.00	0.58 ± 0.57

#### 3.1.2. Plant Biomass

**Figure 2:**
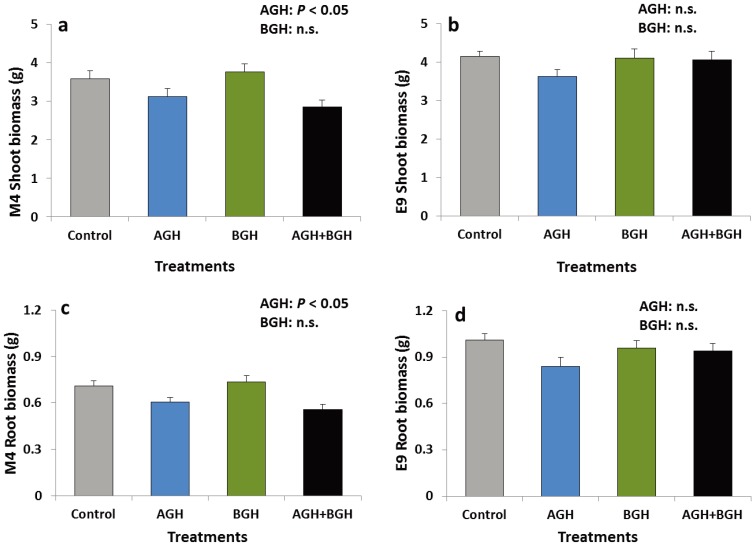
Shoot biomass (g) of the genotype M4 (**a**) and E9 (**b**) and root biomass (g) of the genotype M4 (**c**) and E9 (**d**) (mean ± SE). Treatments: Control, AGH: aboveground herbivory only, BGH: belowground herbivory only, AGH+BGH: aboveground herbivory followed by belowground herbivory with a lag phase of seven days.

In genotype M4, AGH had a significant main effect on the shoot and root biomass (shoots: F [1, 48] = 11.652, *p* = 0.00131; roots: F [1, 48] = 16.16; *p* = 0.0002). The shoot and root biomass were lower on the plants that had received AGH (Figure 2a, c). Neither interaction effects nor a significant main effect of BGH were detected. No treatment effects were detected on the genotype E9 (Figure 2b, d).

The genotype-wise comparisons of the total shoot and root biomass, regardless of the treatments, showed a significant difference between the two genotypes. Genotype E9 had significantly higher shoot (t [102] = 8.83, *p* = 0.005) and root (t [102] = 4.38, *p* < 0.001) biomass than M4; E9 had a shoot biomass of 3.98 ± 0.10 g while M4 weighed 3.33 ± 0.10 g, E9 had a root biomass of 0.93 ± 0.03 g whereas M4 weighed 0.65 ± 0.02 g.

#### 3.1.3. PI and Protein Content in the Root Tissue

**Figure 3:**
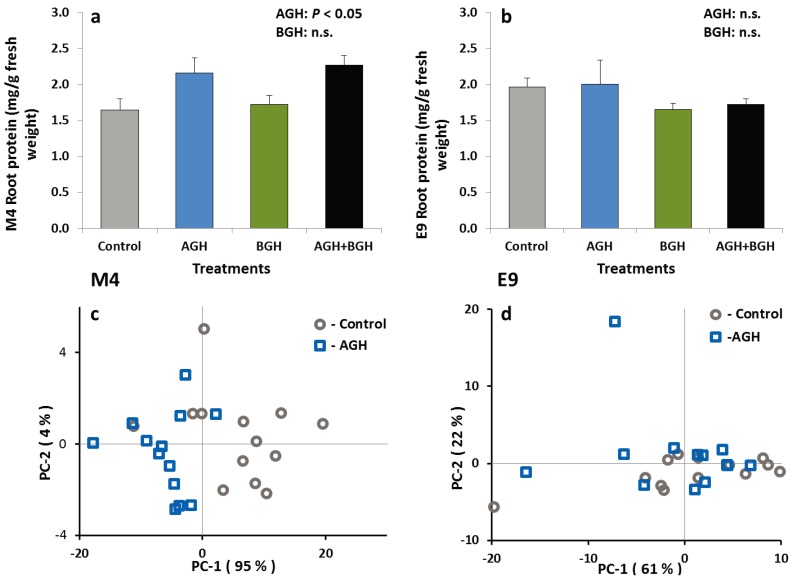
Total protein content (mg/g fresh weight) in the root tissue of the genotype M4 (**a**) and E9 (**b**) (mean ± SE) and results from PCA of the FT-IR spectra of the root material from M4 (**c**) and E9 (**d**) (spectral ranges of 1680–1620 cm^−1^ and 1550–1500 cm^−1^. Spectra are treated by vector normalization). Treatments: control, AGH: aboveground herbivory only, BGH: belowground herbivory only and AGH+BGH: aboveground herbivory followed by belowground herbivory with the lag phase of seven days.

The treatments had no effects on the total PI content in the roots of the plants from either genotype. Similarly, the total protein content in the E9 roots did not differ among herbivory treatments, but there was a significant treatment effect on the total protein content in M4 roots (F [1,48] = 12.02; *p* = 0.0012) (Figure 3a). Root tissues of the M4 plants that received the AGH contained significantly higher levels of protein. There was no interaction between AGH and BGH. These results were supported by PCA of the FT-IR spectra from the root material of both genotypes as shown in Figure 3. Evaluation of characteristic spectral ranges for proteins (amide I and II bands, 1680–1620 cm^−1^ and 1550–1500 cm^−1^) results in a clear separation of the root material from control and AGH plants for the M4 genotype (Figure 3c). In contrast, for E9, PCA of the root material based on amide bands yielded no separation (Figure 3d). 

#### 3.1.4. Carbon and Nitrogen Concentration

**Figure 4:**
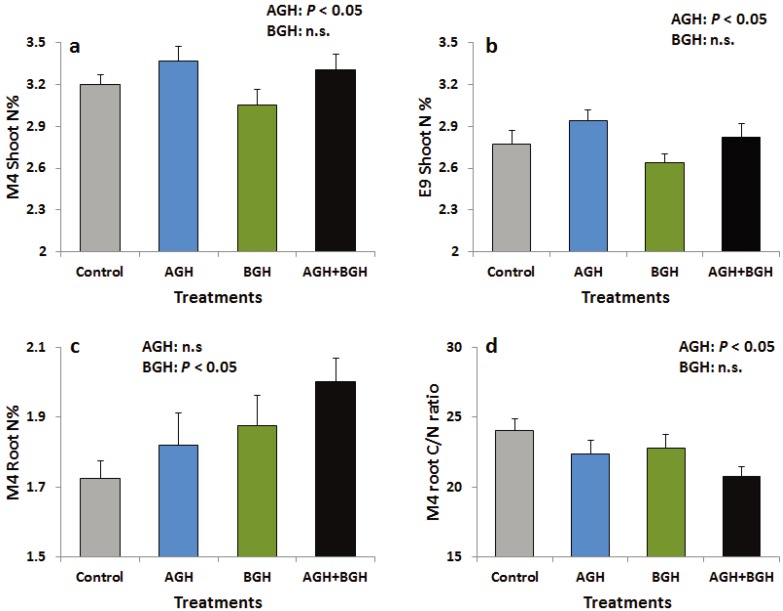
Shoot N (%) of the genotype M4 (**a**) and E9 (**b**), root N (%) of the genotype M4 (**c**) and root C/N ratio of the genotype M4 (**d**). Treatments: Control, AGH: aboveground herbivory only, BGH: belowground herbivory only and AGH+BGH: aboveground herbivory followed by belowground herbivory with the lag phase of seven days.

AGH had significant main effects on the leaf N concentration in both genotypes (Supplementary Table S1; Figure 4a, b). N concentration was increased in the shoots of both genotypes in response to AGH while there were no changes in C concentration and shoot C/N ratio. In M4, AGH significantly decreased the C/N ratio of the root tissue (Supplementary Table S1, Figure 4d) while BGH significantly increased root N concentration (Supplementary Table S1, Figure 4c), but E9 was not affected.

#### 3.1.5. Feeding Bioassay

In the non-choice feeding assay, *Agriotes* larvae ate the root material from all treatments without any significant preference. However, there was a tendency of the larvae to prefer the roots of M4 genotype over E9 when comparing the total amount of roots eaten (F [1, 86] = 3.29; *p* = 0.073) in the non-choice bioassay (Table 2). In the choice bioassay, BGH had a significant negative effect on the amount of root eaten (D [1, 49] = 6.32; *p* = 0.013) in E9 genotype (Table 2).

The weight gain of larvae was increased by BGH when offered M4 plants in the non-choice bioassay (F [1, 40] = 4.49; *p* = 0.0404) (Table 2). In the choice bioassay, the weight gain of larvae was significantly higher on M4 roots than on E9 roots irrespective of treatment (F [1, 24] = 4.33; *p* = 0.048) (Table 2). 

**Table 2:** Total amount of roots eaten in mg (mean ± SE) and weight gain of the larvae in mg (mean ± SE) by *Agriotes* larvae in choice and non-choice feeding bioassay.

Bioassay	Genotypes	Root eaten (mg)				Aggregate
		Control	AGH	BGH	AGH+BGH	
Non-Choice	M4	3.6 ± 0.20	3.62 ± 0.08	3.73 ± 0.11	3.53 ± 0.18	3.62 ± 0.07
	E9	3.57 ± 0.15	3.55 ± 0.17	3.27 ± 0.09	3.33 ± 0.14	3.43 ± 0.07
Choice	M4	2.03 ± 0.16	2.22 ± 0.16	1.95 ± 0.12	2.03 ± 0.09	2.06 ± 0.07
	E9	2.20 ± 0.10	2.21 ± 0.19	1.78 ± 0.19	1.85 ± 0.08	2.01 ± 0.08
**Bioassay**	**Genotypes**	**Weight gain of the larvae (mg)**				
		**Control**	**AGH**	**BGH**	**AGH+BGH**	
Non-Choice	M4	0.43 ± 0.14	0.46 ± 0.16	1.17 ± 0.35	0.79 ± 0.30	0.71 ± 0.13
	E9	0.31 ± 0.24	0.85 ± 0.19	0.45 ± 0.21	0.33 ± 0.24	0.48 ± 0.11
Choice		**M4**		**E9**		
		1.59 ± 0.41		0.51 ± 0.32		

### 3.2. Discussion

The results from this experiment clearly demonstrate that previous AG herbivory induces genotype-specific changes in quantitative and qualitative root traits which may have consequences for belowground interactions including herbivory. These changes were found only in the genotype M4 but not in the genotype E9 of *S. dulcamara*. However, the changes in plant traits due to AG herbivory did not have any effects on the performance of a BG generalist herbivore in our model system. 

#### 3.2.1. AG Herbivory Effects on Plant Traits

In this experiment, AGH had significant effects on shoot and root biomass, and the nutritive value of *S. dulcamara.* As the roots are responsible for nutrient allocation, reduced root biomass due to AGH may limit nutrient uptake capacity of the plant and at the same time affect the plant’s interactions with herbivores. Primary compounds which play key roles in the physiological processes of the plant also have significant effects on plant–herbivore interactions. Nitrogen is a very important plant nutrient which can limit the growth and performance of herbivores [48]. In the shoot of both genotypes and the root of M4, the N concentration was increased in response to AGH and BGH, respectively. Huang *et al.* [49] found elevated levels of N and increased larval survival of the chrysomelid *Bikasha collaris* in the root tissue of *Triadica sebifera* (*Euphorbiaceae*) plants due to AGH by conspecific adults. Consistently, in our feeding bioassay, we found a higher weight gain of *Agriotes* larvae on M4 roots after belowground herbivory by conspecifics that also enhanced the nitrogen concentration in M4 roots. However, previous aboveground herbivory did not change the larval growth of *Agriotes* in the feeding bioassays. AGH reduced the C/N ratio of the root tissue of M4 plants, which indicates a relative increase of N compared to C or a relative decrease of C compared to N. Further, AGH increased protein levels in M4 roots suggesting rather a relative increase of N compared to C, because N is a principal component of proteins. In addition, the choice and non-choice bioassays indicated that the root of the M4 genotype had a better food quality as the *Agriotes* larvae tended to eat more roots from M4 plants in non-choice bioassay, and the weight gain was higher in M4 root compared to E9 roots. Increased levels of nitrogen and protein in the root may promote these effects. 

Previous studies have shown that *S. dulcamara* is able to induce defense compounds such as polyphenol oxidase and PIs in response to AG herbivory [50,51]. PIs are a large group of plant proteins which may function as natural defensive compounds against herbivores [52]. Based on the previous results of PI induction in leaves of *S. dulcamara* plants [51], we were interested to see if PIs are induced in the root tissue in response to previous shoot or root herbivory but we found no significant changes in PI levels. In a study by Yang *et al.* [53], it was found that AG herbivory by whiteflies (*Bemisia tabaci*) in pepper (*Capsicum annuum*) induces the upregulation of transcriptional expression of *Capsicum annuum protease inhibitor II (CaPIN II)* genes in the roots. 

#### 3.2.2. Effects of Changes in Plant Traits by AG Herbivory on BG Herbivores

In a study using *S. dulcamara* as a model plant, it was found that previous leaf herbivory by the chrysomelid beetle *Psylliodes affinis* negatively influenced the performance of later feeding leaf herbivores, both conspecifics and the beetle *Plagiometriona clavata* [35]. These effects persisted even in the next generation where the emergence of second-generation *P. clavata* adults on the plants was decreased. In contrast, no effects of previous AGH by *S. exigua* on the performance of later feeding BG herbivores were found in our experiment. The reason could be associated with the herbivore identity. Viswanathan *et al.* [35] found plant-mediated interactions among the specialist Coleopteran species, while we used a generalist Lepidopteran AG herbivore and a generalist Coleopteran BG herbivore in our experiment. The type of interacting herbivores is an important factor for the outcome of AG and BG herbivore interactions [9]. Further, *Agriotes* larvae may not be affected by changes in root quality and quantity if there is enough root material available for feeding as suggested by Sonnemann *et al.* [37]. As *S. dulcamara* provided enough root material for *Agriotes* larvae, the changes in the relative proportion of primary metabolites and total protein had no significant effects on them. Lack of increase in larval weight is consistent with a previous study by Sonnemann *et al.* [37] where larvae were allowed to feed on the root system for a longer period (12 days) than in this experiment (7 days). *Agriotes* larvae have a long life cycle of about two years [54] and do not grow very quickly. Thus, subtle changes in their performance during our experimental period of seven days might not be discernible. Therefore, no history effects of the previous AG herbivory were detected on the BG herbivore performance. However, we detected an effect of genotype identity on the weight gain of the larvae in the bioassays. The larvae tended to eat more and gained more weight on M4 roots in the bioassays suggesting a higher nutritive quality of this genotype. The detection of the effect only in the bioassays might be related to the fact that the root mass offered in the bioassays was more limited than in the greenhouse pot experiment. According to Erb *et al.* [8], AG herbivores have negative effects on BG herbivores when added first to the plant; but a recent meta-analysis documented that such effects were mainly found in annual plants but not in perennial plant species [9]. We also suspect that the density of BG herbivore individuals in the root tissue was likely not enough to trigger a significant response from the plants. Even though root biomass is a limiting factor for the responsiveness of *Agriotes* larvae, the reduced root biomass in response to AGH was still sufficient for the density of *Agriotes* larvae and the duration of root herbivory used in this experiment.

#### 3.2.3. Differences among the Plant Genotypes

The effects of AGH on plant characteristics were clearly genotype dependent. Genotype M4 was found to be more responsive to herbivory than E9 where only shoot C/N ratio was affected by AGH. The difference in the feeding behavior of the AG herbivore on the leaves of the two genotypes may be related to the relative nutritive value and/or defense status of the leaves. In addition, the weight gain by BG herbivores feeding on M4 root material was higher in the choice bioassay, suggesting also differences in quality of the different genotypes. This hypothesis is well supported by the results from FT-IR analysis which was carried out to estimate the differences in metabolic profiles of both plant genotypes and changes due to the herbivory. The results from FT-IR analysis showed significant differences in the metabolic profile of leaves and roots of M4 and E9. Additionally, differences in metabolic profiles as a consequence of AG herbivory were detected for the roots of M4. The M4 plants have lower AG and BG biomass than E9, thus they might be more responsive to protect their limited plant parts and resources. Similarly, genotype-dependent responses of plant species have been shown in a few previous studies. For example, Wurst *et al.* [33] found that the genetic variation in the plant species *Plantago lanceolata* led to dissimilar defense responses in leaves when challenged with BG *Agriotes* larvae, and the subsequent AG herbivory damage also differed between the genotypes. Likewise, in another study, Hol *et al.* [32] showed that the allocation of the defense compound pyrrolizidine alkaloids (PAs) of *Senecio jacobaea* plants to the roots and shoots in response to leaf herbivory by *Mamestra brassicae* was genotype dependent. 

## 4. Conclusions

In summary, our results demonstrate that previous aboveground herbivory can have genotype-specific effects on quantitative and qualitative root traits. It is likely that these changes in the root system have consequences for belowground interactions such as plant–root herbivore interactions. However, these changes might not have any effect on BG herbivores which are insensitive to such changes. Genotypic variability of the model plant should be taken into account in future ecological studies of plant mediated interaction between above- and belowground herbivores, because the results may be genotype- rather than plant species-specific. 

## Supplementary Files

Supplementary File 1
