# Supplementary material for: Genetic Variation of the Host Plant Species Matters for Interactions with Above- and Belowground Herbivores
Source: Insects. 2014 Aug 29;5(3):651–67. doi: 10.3390/insects5030651 (PMC4592585; doi:10.3390/insects5030651)
Supplement: Supplementary File 1 [file insects-05-00651-s001.pdf]

Supplementary Material

Table S1. Two-Way ANOVAs for shoot and root nitrogen and C/N ratio in genotype M4 and E9.

		df	***F***	***p***
M4	*Leaf N*			
	AGH	1	4.347	**0.0424**
	BGH	1	1.08	0.3039
	AGH*BGH	1	0.197	0.6589
	Leaf C/N Ratio			
	AGH	1	3.37	0.0726
	BGH	1	0.782	0.381
	AGH*BGH	1	0.151	0.6995
	Root N			
	AGH	1	2.187	0.1457
	BGH	1	4.834	**0.0328**
	AGH*BGH	1	0.043	0.8361
	Root C/N Ratio			
	AGH	1	4.467	**0.0398**
	BGH	1	2.666	0.109
	AGH*BGH	1	0.062	0.8045
E9	*Leaf N*			
	AGH	1	4.172	**0.0466**
	BGH	1	2.32	0.1343
	AGH*BGH	1	0.008	0.9285
	Leaf C/N Ratio			
	AGH	1	1.2437	0.2703
	BGH	1	2.817	0.0998
	AGH*BGH	1	0.1513	0.699
	Root N			
	AGH	1	1.445	0.2352
	BGH	1	0.4572	0.5022
	AGH*BGH	1	1.1856	0.2817
	Root C/N Ratio			
	AGH	1	1.44	0.236
	BGH	1	0.4735	0.4947
	AGH*BGH	1	1.1117	0.297

Figure S1. Principle component analysis of the FT-IR spectra from the dried and ground root material for M4 (green) and E9 (orange) over the spectral range of 3600–375 cm^{-1}. Spectra are treated by first derivative and vector normalization.

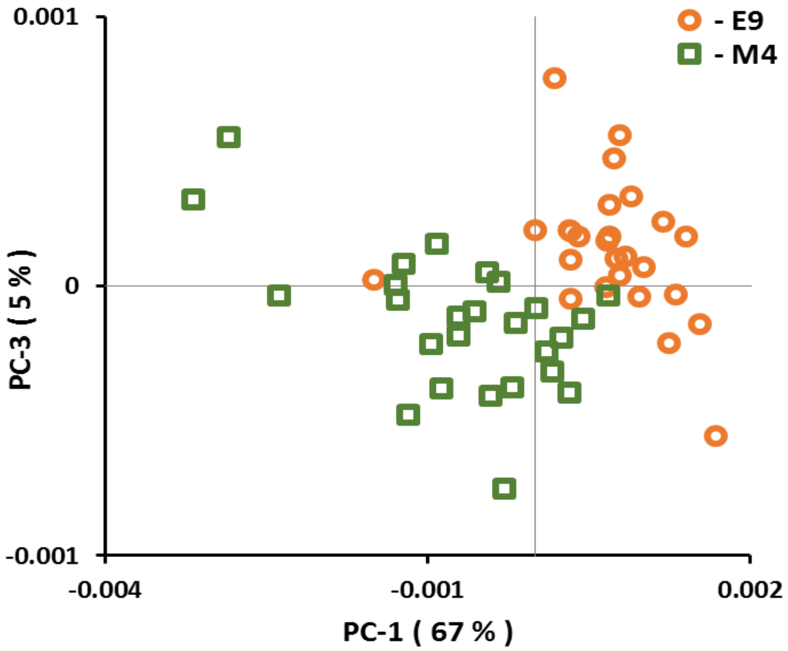

Figure S2. Averaged FT-IR spectra for the leaf material (**a**) and root material (**b**) of M4 (green) and E9 (orange). The dotted lines show the corresponding standard deviation indicating most variation between the genotypes for proteins (amid bands around 1600 cm^{-1}) and carbohydrates (1100–1000 cm^{-1}).

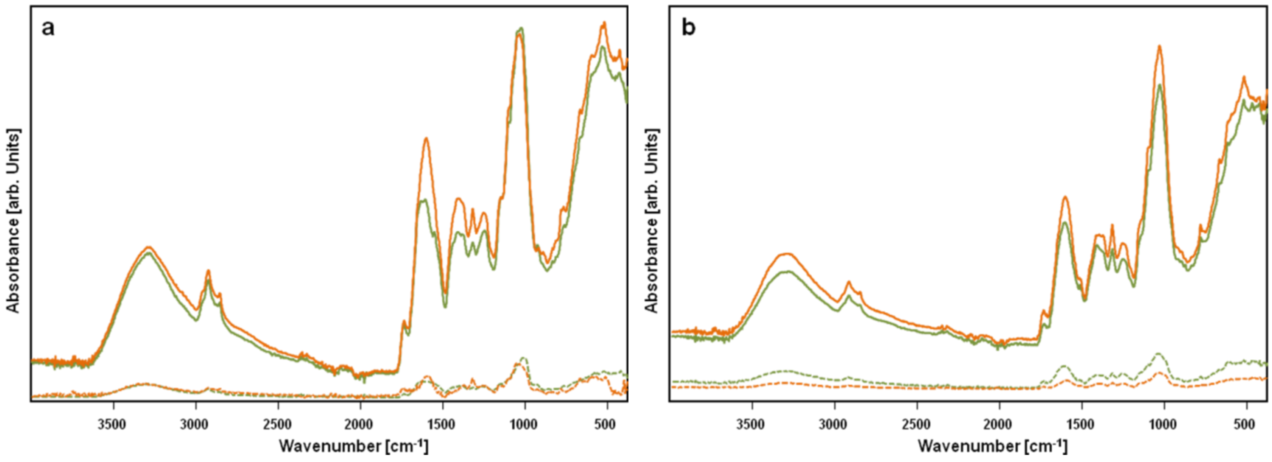

© 2014 by the authors; licensee MDPI, Basel, Switzerland. This article is an open access article distributed under the terms and conditions of the Creative Commons Attribution license (http://creativecommons.org/licenses/by/3.0/).
